# Neuroadaptation in Nicotine Addiction: Update on the Sensitization-Homeostasis Model

**DOI:** 10.3390/brainsci2040523

**Published:** 2012-10-17

**Authors:** Joseph R. DiFranza, Wei Huang, Jean King

**Affiliations:** 1 Department of Family Medicine and Community Health, University of Massachusetts Medical School, 55 Lake Avenue, Worcester, MA 01655, USA; 2 Department of Psychiatry, University of Massachusetts Medical School, 55 Lake Avenue, Worcester, MA 01655, USA; Email: wei.huang2@umassmed.edu (W.H.); jean.king@umassmed.edu (J.K.)

**Keywords:** tobacco, nicotine, addiction, dependence, sensitization, homeostasis, neuroadaptation

## Abstract

The role of neuronal plasticity in supporting the addictive state has generated much research and some conceptual theories. One such theory, the sensitization-homeostasis (SH) model, postulates that nicotine suppresses craving circuits, and this triggers the development of homeostatic adaptations that autonomously support craving. Based on clinical studies, the SH model predicts the existence of three distinct forms of neuroplasticity that are responsible for withdrawal, tolerance and the resolution of withdrawal. Over the past decade, many controversial aspects of the SH model have become well established by the literature, while some details have been disproven. Here we update the model based on new studies showing that nicotine dependence develops through a set sequence of symptoms in all smokers, and that the latency to withdrawal, the time it takes for withdrawal symptoms to appear during abstinence, is initially very long but shortens by several orders of magnitude over time. We conclude by outlining directions for future research based on the updated model, and commenting on how new experimental studies can gain from the framework put forth in the SH model.

## 1. Introduction

The proposition that neuroadaptation causes nicotine addiction implies that neural alterations caused by smoking represent more than nonspecific drug toxicity [1]. Addiction theories are challenged to explain why a drug triggers neuroadaptation and how neuroadaptation causes addiction. The operative paradigm for generations of addiction researchers has been the theory that drugs shape behavior and cause addiction through reinforcement, a process that would not appear to require neuroadaptation beyond that which might be involved in conditioned learning. In contrast to the reinforcement paradigm, the sensitization-homeostasis (SH) model postulated that the development and recovery from nicotine addiction reflects 3 distinct neuroadaptive processes that appear unrelated to reward and reinforcement [2]. Thus, the SH model offered a distinctly new framework for conceptualizing the role of neuroadaptation in addiction. Nearly a decade has passed since the publication of the SH model [2,3]. Here we critique and update the model in light of new research and outline theory-based directions for future research. 

## 2. The Major Principles of the Sensitization-Homeostasis Model

**Figure 1:**
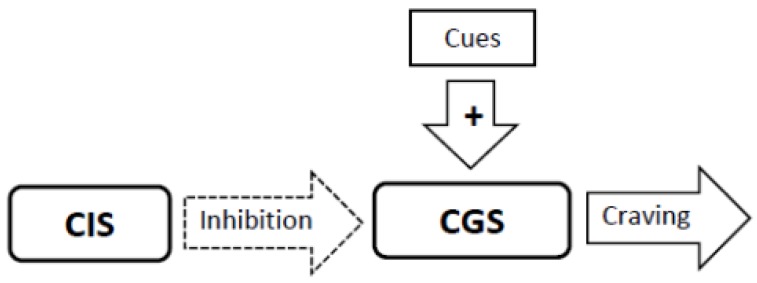
The Sensitization Homeostasis Model. The Craving Generation System (CGS) is a neural network that is responsible for generating craving for substances or experiences (the output from which is represented by the arrow labeled craving). The Craving Inhibition System (CIS) signals satiation by inhibiting the CGS. Smoking cues can stimulate craving by stimulating the CGS. The dashed outline on the inhibitory arrow indicates that the CIS system is inactive.

**Figure 2:**
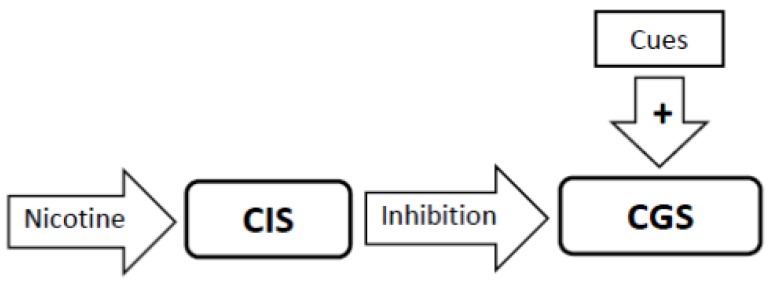
The primary addiction-related action of nicotine is to stimulate the Craving Inhibition System (CIS) which is experienced as a sense of satisfaction by the smoker. Stimulation of the CIS results in inhibition of the Craving Generation System (CGS) as indicated by the solid inhibitory arrow. This effect can block the ability of smoking cues to generate craving.

**Figure 3:**
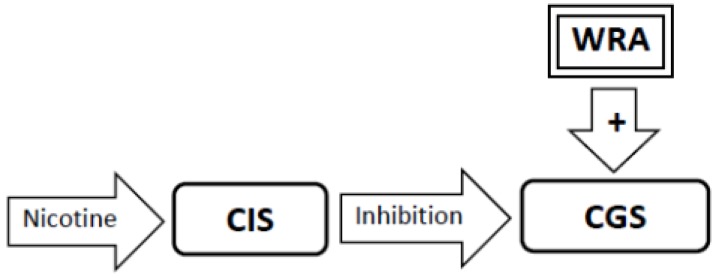
As a result of sensitization to nicotine, the inhibition of the Craving Generation System (CGS) produced by nicotine is super-physiologic. The excessive inhibition of the CGS caused by nicotine disrupts homeostasis in the CGS. This prompts the rapid development of Withdrawal-Related Adaptations (WRA) that stimulate the CGS to restore homeostasis.

**Figure 4:**
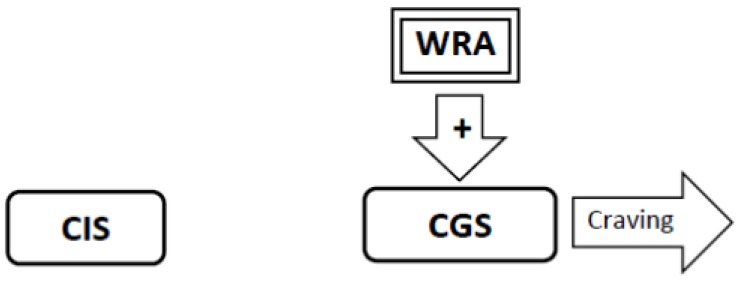
When the down-stream effects of nicotine wear off, the Withdrawal-Related Adaptations (WRA) continue to stimulate the Craving Generation System (CGS) resulting in the spontaneous generation of craving in the absence of smoking cues. This prompts the smoker to smoke every time the effect of nicotine wears off.

**Figure 5:**
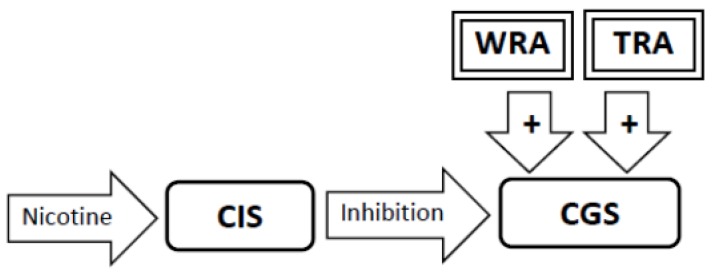
Repeated exposures to nicotine promote additional neuroadaptation. The Withdrawal-Related Adaptations (WRA) are bolstered by Tolerance-Related Adaptations (TRA). The TRA also stimulate the Craving Generation System (CGS). TRA have the effect of shortening the latency to the onset of withdrawal-related craving. As TRAs develop smokers notice that the latency to the onset of withdrawal-related craving may shorten from weeks to days, to hours, and in some cases, to minutes. The WRAs and TRAs do not directly block the effect of nicotine as the nicotine from one cigarette is sufficient to alleviate withdrawal-related craving whether a person has been smoking for a few weeks or many decades.

**Figure 6:**
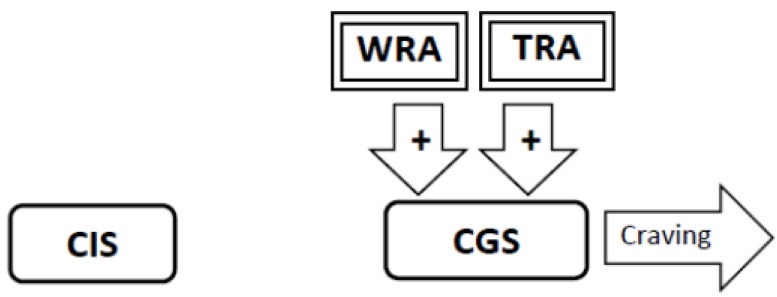
When cessation is attempted, the Withdrawal-Related Adaptations (WRA) and Tolerance-Related Adaptations (TRA) continue to stimulate the Craving Generation System (CGS) resulting in withdrawal-related craving in the absence of any cues. Now the stimulation of the CGS provided by the WRA and TRA is no longer restoring homeostasis but is disrupting homeostasis. To restore homeostasis the brain removes the WRA.

**Figure 7:**
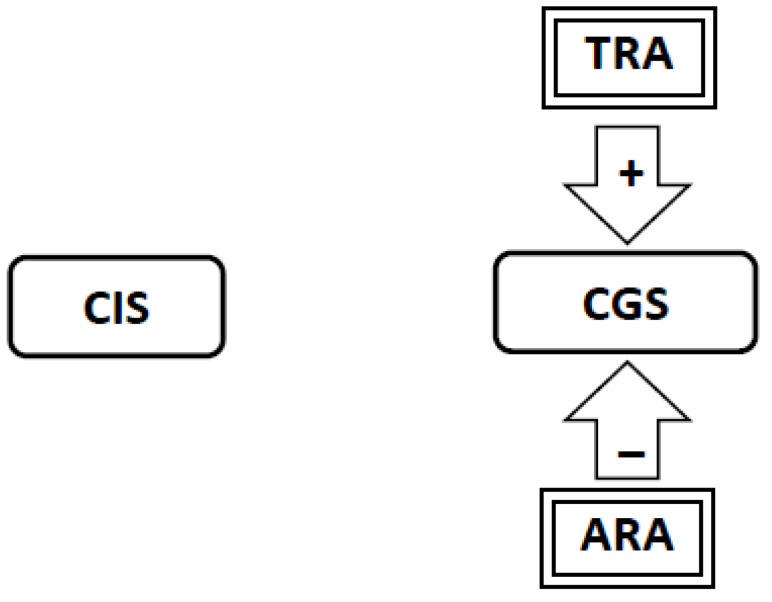
After smoking cessation, the brain dismantles the Withdrawal-Related Adaptations but is unable to dismantle the Tolerance-Related Adaptations (TRA). Unless something is done to re-establish homeostasis, craving would continue forever. To restore homeostasis, Abstinence-Related Adaptations (ARA) develop to provide inhibitory input to the Craving Generation System (CGS) counter-balancing the stimulatory input from the TRA. At this point, withdrawal-related craving ends, but craving could still be stimulated by smoking or emotional cues.

**Figure 8:**
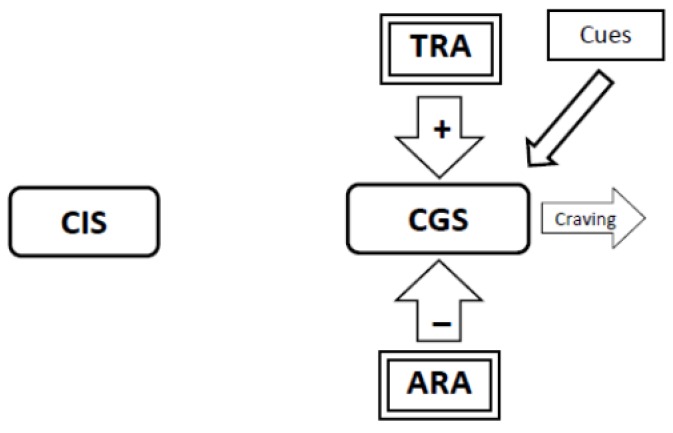
At any time cues could trigger minor craving by directly stimulating the Craving Generation System (CGS). The smoker might lapse by smoking a single cigarette.

**Figure 9:**
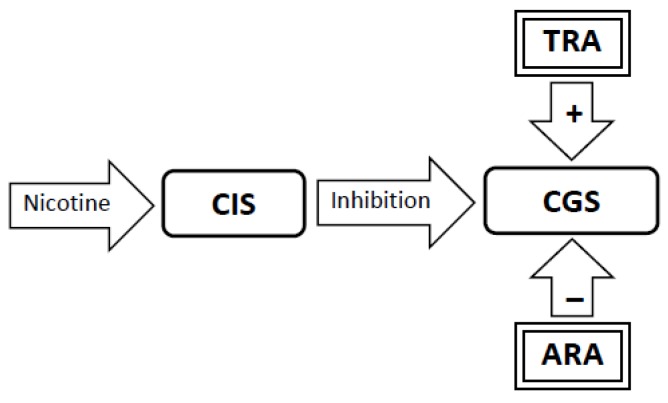
When an ex-smoker lapses and smokes a cigarette, nicotine stimulates the Craving Inhibition System (CIS) which delivers super-physiologic inhibition to the Craving Generation System (CGS), once again disrupting homeostasis in the CGS. The Abstinence-Related Adaptations (ARA) that provide inhibition to the CGS are now adding to the disruption of homeostasis rather than restoring it. The ARA are rapidly dismantled.

**Figure 10:**
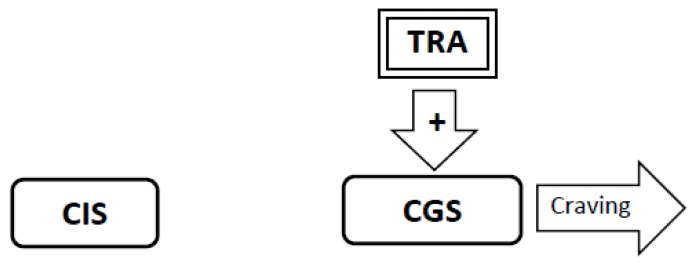
With the Abstinence-Related Adaptations (ARA) gone, when the down-stream effects of nicotine wear off, the Tolerance-Related Adaptations (TRA) provide un-opposed stimulation to the Craving Generation System (CGS) and this restores craving to its original intensity. Because the Tolerance-Related Adaptations set the latency to the onset of withdrawal craving, relapsed smokers find that they need to smoke at nearly the same frequency as when they had quit smoking no matter how long they had been abstinent.

1. The brain has two neural networks, a Craving Generation System and a Craving Inhibition System. The Craving Generation System is responsible for motivating appetitive behaviors, while the Craving Inhibition System quiets the Craving Generation System when satiety is achieved (Figure 1).

2. Nicotine’s direct action is to activate the Craving Inhibition System, resulting in feelings of satisfaction and relaxation. When activated, the Craving Inhibition System suppresses activity in the Craving Generation System, thereby inhibiting craving. The nicotine-induced activation of the Craving Inhibition System and subsequent suppression of the Craving Generation System exceed that which would occur with satiety to non-drug appetites (Figure 2).

3. Excessive and prolonged inhibition of the Craving Generation System by nicotine prompts the rapid development of stimulatory *withdrawal-related adaptations* that counter nicotine’s effects. These restore activity in the Craving Generation System to normal despite the lingering inhibitory effect of nicotine (Figure 3).

4. When the indirect inhibitory effect of nicotine wears off, the stimulatory withdrawal-related adaptations activate the Craving Generation System. This causes the addicted smoker to experience withdrawal-induced craving for nicotine whenever the effect of nicotine wears off (Figure 4). In novice smokers, it may take several weeks for the down-stream effects of nicotine to wear off, and consequently the latency from the last cigarette to the onset of withdrawal-related craving may be several weeks.

5. Repeated exposures to nicotine promote additional neuroadaptation. The *withdrawal-related adaptations* are joined by *tolerance-related adaptations* that also stimulate the Craving Generation System (Figure 5). The *tolerance-related adaptations* have the effect of shortening the latency to the onset of withdrawal-related craving.

6. When cessation is attempted, the *withdrawal-related adaptations* and *tolerance-related adaptations* continue to stimulate the Craving Generation System resulting in withdrawal-related craving in the absence of any cues (Figure 6). 

7. The *withdrawal* and *tolerance-related adaptations* are now disrupting homeostasis. In an attempt to restore homeostasis the brain removes the *withdrawal-related adaptations* but cannot remove the *tolerance-related adaptations*. To restore homeostasis, *abstinence-related adaptations* develop to provide inhibitory input to the Craving Generation System counter-balancing the stimulatory input from the persistent *tolerance-related adaptations* (Figure 7).

8. The model also explains why smoking a single cigarette so often causes a relapse (Figure 8, Figure 9, Figure 10).

## 3. A Review of Recent Literature in Relation to the Sensitization-Homeostasis Model

In this section we will describe aspects of the SH model (in italics) in relation to recent research [2,3]. 

### 3.1. The SH Model Holds that the Neural Processes that Produce Addiction Are Initiated Soon after the Onset of Nondaily Smoking, Stipulating that Addiction Is Possible in the Absence of Sustained Moderate Daily Smoking

Early symptoms of addiction have now been documented soon after the onset of nondaily smoking in every study that has examined this issue [4,5,6,7,8,9,10,11,12,13,14]. The experience of withdrawal-induced craving after having smoked only a few cigarettes is also well documented [5,10,15,16]. In the largest study of adolescent nicotine addiction ever conducted, early symptoms of addiction were reported by a third of youth who had smoked 3 or 4 cigarettes, and in about 95% of those who had smoked ≥100 cigarettes [10]. When measured by the DSM diagnostic criteria for nicotine dependence, dependence has been identified prior to the onset of daily smoking, and in individuals who had not yet smoked 12 cigarettes [5,17].

Although the clinical significance of early appearing symptoms of addiction had been questioned, they have proven to be powerful predictors of the clinical course. In a 3-year prospective study, youth who reported such symptoms were 44-fold more likely to be current smokers at the end of follow-up [6]. In another study, youth that reported at least one symptom of addiction were 196-fold more likely to progress to daily smoking [7]. 

The clinical importance of early symptoms is indirectly supported by genetic studies. “The genetic overlap that was found between experimentation and withdrawal is important in the context of dependence syndrome models that attribute a key role to withdrawal symptoms in the emergence of dependence. This result raises the possibility that at least some individuals become ‘hooked’ or progress to daily smoking in part because of increased vulnerability to nicotine withdrawal symptoms early in their smoking careers [18]”. Thus, the available evidence indicates that symptoms of addiction appear soon after the onset of nondaily use in the most vulnerable individuals, and that early symptoms are strong predictors of continued use.

### 3.2. The SH Model Stipulated that Neuroadaptations Develop Quickly with Intermittent Nicotine Exposures

The original paper describing the SH model cited the neuroscience literature extensively to demonstrate the scientific plausibility of the idea that small doses of nicotine could change the brain. In doing so, the model reflected the then current thinking in regard to nicotinic receptor upregulation and desensitization. Much of that speculation is now dated as continued research suggests that upregulation and desensitization of receptors is a much more transient and dynamic process than previously appreciated [19,20]. As a consequence, the proposal that addicted smokers are able to sustain a pattern of nondaily smoking because of persistent upregulation of AChRs seems unlikely. Nevertheless, the literature provides ample new evidence supporting the principle that small doses of nicotine can change the brain. 

Noting that an average smoker might smoke 10 cigarettes in a day, common sense might suggest that the nicotine delivered by a single cigarette is negligible, and that it is implausible that one cigarette could have a permanent impact on the brain. Yet, a PET study revealed that the nicotine obtained from 2 puffs of cigarette smoke was sufficient to occupy 50% of the brain’s high affinity α4β2 nicotinic receptors (nAChR), while the nicotine from a whole cigarette occupied 88% of nAChRs [21]. This lends plausibility to the idea that one cigarette might have an important impact.

The neuroplastic changes that are specifically responsible for nicotine addiction have not been identified. However, it is becoming increasingly clear that even intermittent doses of nicotine can have immediate and sometimes enduring effects of brain physiology and animal behavior. In a direct test of the SH model’s prediction that one cigarette can initiate neural changes, Slotkin *et al.* demonstrated nAChR up-regulation in rats within 24 h of a single dose of nicotine [22,23]. More recent work indicates that receptor upregulation begins within minutes of an exposure to nicotine [19].

Rapid changes in neurophysiology after a single exposure to nicotine have been seen in other studies. In mice, a single dose of nicotine produces an increase in the intracellular AMPAR/NMDAR ratio in neurons within 24 h [24]. Using mecamylamine precipitated withdrawal as a discriminative cue, Cohen *et al*. demonstrated that rats can sense withdrawal symptoms after a single dose of nicotine [25]. 

One long-term effect of nicotine that is seen with the first dose is a lowering of neural response thresholds. When response thresholds are lower, neurons become responsive to lower levels of stimulatory inputs. Placzek *et al.* reported that a nicotine-induced increase in either spontaneous or weakly evoked excitatory currents persists after nicotine is removed from the bath solution, suggesting the induction of long-term potentiation [24]. Mao *et al*. have recently described the pathway of intracellular events involved in synaptic plasticity and long-term potentiation that result from a single exposure to nicotine [26]. Synaptic plasticity developed after the nicotine was removed [26]. Hamid *et al.* demonstrated that a single dose of nicotine in naïve rats lowers neural response thresholds for 28 days [27]. These studies demonstrate that a single exposure to nicotine sets off a series of intracellular events that proceed of their own accord after nicotine is gone, with objectively measurable effects on neural function up to 28 days later.

Magnetic resonance imaging (MRI) studies demonstrate that smokers have increased structural complexity in white matter tracts [28,29,30]. Animal studies demonstrate how quickly these changes can develop. MRI scans obtained after rats had received 4 doses of nicotine revealed a significant increase in tissue density in the cingulate cortex and a trend in the same direction in the nucleus accumbens and prefrontal cortex [31]. 

Neuroplastic changes can be observed directly as in the above studies, or can be inferred from behavioral studies. Acute tolerance to the locomotor depressant effects of nicotine are evident after a single dose in rats [32]. One dose of nicotine produces behavioral effects in adolescent rats that are present for a month [33,34]. Locomotor sensitization to nicotine begins with the first dose, as increased locomotion is commonly observed with the second dose [35,36]. Locomotor sensitization is accompanied by changes in neural responses to nicotine. A dose of nicotine delivered to sensitized rats produces neural activation that is more widespread, more prolonged and of greater magnitude than that seen in nicotine naïve animals [37]. Functional MRI demonstrates changes in neural responses to nicotine after 4 doses [37]. 

An intriguing study by Polesskaya *et al*. suggests why a single exposure to nicotine can trigger long-lasting changes [38]. Nicotine delivered through an implantable pump altered the expression of 162 genes in the brains of adolescent rats. As the investigators studied only 38% of the genome, the actual number of genes affected by nicotine may be close to 500. Widespread alterations in gene expression would provide a plausible mechanism by which a single exposure to nicotine could affect brain physiology immediately and persistently.

The studies reviewed in this section do not prove that the neuroplastic changes that are responsible for nicotine addiction begin with the first dose, but they do indicate that even brief intermittent exposures to nicotine trigger changes in brain physiology, structure and function, along with changes in behavioral responses that persist long after nicotine is gone from the brain. Parenthetically, many of these changes were observed in experiments in which learning and reinforcement could play no role (cell preparations, non-contingent nicotine administration). 

### 3.3. Nicotine Withdrawal Is Present in Nondaily Smokers. This Contradicted the Prevailing Paradigm that Smokers Do Not Experience Withdrawal or Addiction Until They Are Smoking at Least 5 Cigarettes per Day [39,40]

Withdrawal-induced craving is a very common presenting symptom of nicotine addiction in adolescents [41]. The first prospective study of the onset of nicotine addiction reported that two-thirds of the individuals that developed symptoms of addiction did so without smoking daily [6,9]. Addiction as defined by the Diagnostic and Statistical Manual-IV criteria has been diagnosed soon after the onset of smoking in nondaily smokers [5]. Addiction in nondaily smokers has been documented in several longitudinal studies [4,5,6,7,8], in cross-sectional studies [10,11,12,13,42,43,44,45], and by case histories [46]. Collectively, these studies have involved tens of thousands of smokers from adolescence to adulthood.

The clinical significance of addiction symptoms reported by nondaily smokers is evident: adult nondaily smokers are just as likely as daily smokers to have failed at a previous attempt to quit [47]. Adolescent nondaily smokers reported an average of 2 prior unsuccessful quit attempts [45]. Early symptoms of addiction have proven to be an excellent predictor of the subsequent clinical course of smoking [48]. In adolescents, the mean frequency of smoking at the onset of addiction is 2 cigarettes per week [6,7], and smoking 2 cigarettes per week at age 12 increases the likelihood that the child will be a heavy smoker at age 24 with an odds ratio of 174 [49]. 

Some older studies with small, non-representative samples reported that smokers who do not smoke at least 5 cigarettes per day had no symptoms of addiction [39,40]. Looking back, these studies could not exclude the possibility of addiction because they did not employ the sensitive measures of dependence now in use. Symptoms of addiction, including those of nicotine withdrawal have been reported among nondaily smokers in every modern study that has examined this issue.

### 3.4. Experimental Conditions that Stimulate Craving Will Increase Neural Activity in the Craving Generation System (Figure 1)

Dozens of functional MRI experiments have examined neural activity that accompanies craving in drug addicted individuals. As neural activity reflects not only craving, but also the processing of the cues that are used to stimulate craving, the observed patterns of activation differ depending upon the experimental conditions. Nevertheless, craving in response to drug cues is fairly consistently associated with activation in the anterior cingulate cortex (ACC) and frontal cortex. Activation of the ACC has been demonstrated in conjunction with opiate craving [50], and exposure to cues for smoking [51,52,53,54,55,56,57,58] and cocaine [59,60,61,62,63]. Self-rated severity of addiction in adolescent smokers correlated (*r* = 0.61) with reactivity to smoking cues in the ACC [57]. This literature suggests that the ACC may be part of a craving generation neural network that is activated during craving for nicotine. 

Bupropion is a drug that is used to reduce craving during nicotine withdrawal. Brody *et al.* demonstrated attenuation of cue-induced craving and ACC activation in bupropion-treated smokers [64]. They also found increased activation in the ACC when smokers were instructed to resist craving [54]. Beyond the fact that this study supports involvement of the ACC in craving, the results are difficult to interpret since the instructions to resist craving might prompt subjects to work up some craving in order to resist it. It is not obvious whether such instructions should prompt an increase or decrease in activity in a neural craving network. Nevertheless, many studies involving different drugs support the idea that neural networks support craving. 

The idea that craving involves a network of neural systems is not unique to the SH model. What is unique to the SH model is the stipulation that cues and nicotine withdrawal will both increase neural activity in a network that supports craving while nicotine administration will have the opposite effect. While drugs such as cocaine may stimulate craving and binging, the SH model stipulates that nicotine inhibits activity in the craving network. Given what is known today about the widespread effects that nicotine has on the brain, it is no longer surprising that nicotine has inhibitory effects, even though it is considered a psychostimulant drug [20].

### 3.5. Nicotine Administration Will Inhibit Activity in the Craving Generation System (Figure 2)

As the SH model holds that nicotine stimulates the Craving Inhibition System which in turn inhibits activity in the Craving Generation System, a critical test of principle would be to determine if nicotine can simultaneously stimulate and inhibit neural activity in different brain regions. Resting state functional connectivity (rsFC) measures the degree to which neural activity in different brain regions is coordinated. Tanabe *et al*. demonstrated that nicotine decreased rsFC in the medial prefrontal cortex and precuneus in nonsmokers [65]. This is an important observation as the subjects had no prior exposure to nicotine and therefore, the reduction in coordinated neural activity triggered by nicotine could not be attributed to the relief of withdrawal symptoms or conditioning effects as might be the case in smokers. Nicotine produced a reduction in coordinated neural activity in nonsmokers in structures that have been shown in other studies to be implicated in nicotine addiction. 

Domino *et al*. conducted an experiment in which PET scans were performed while smokers smoked after an overnight abstinence [66]. The SH model indicates that the Craving Generation System would be active after overnight withdrawal in daily smokers. Under these conditions, smoking would activate the Craving Inhibition System causing inhibition of the Craving Generation System. In the experiment, smoking produced a large increase in regional cerebral blood flow (rCBF) in the cerebellum, occipital cortex, and insula, accompanied by large decreases in rCBF in the ACC, orbitofrontal cortex, nucleus accumbens, fusiform gyrus, hippocampus and the parietal cortex [66]. To our knowledge, this was the first study to show inhibitory effects of nicotine on neural activity. 

The main findings of Domino *et al.* were replicated in another PET study in which rCBF was measured in abstinent smokers while they smoked after an overnight withdrawal [67]. rCBF decreased in response to smoking in the ACC, prefrontal cortex, nucleus accumbens, amygdala and hippocampus. Decreased activity in the left dorsal ACC and the right hippocampus correlated with a decrease in craving scores. The regional specificity of the effects, the bidirectional results, the correlation with craving, and the fact that the regional patterns do not correspond to anatomical areas of vascular supply make it highly implausible that the results of these PET studies reflect direct vasoactive effects of nicotine.

As rsFC relies on correlations in the frequency of activity in different brain regions, it is not susceptible to vasoactive effects. Wang *et al.* compared rsFC in smokers in withdrawal after a 12 h abstinence and the same subjects after they had smoked a cigarette [68]. Craving during withdrawal correlated with increased rsFC in the ACC, dorsal-lateral prefrontal cortex, orbital-frontal cortex, occipital cortex, ventral striatum, nucleus accumbens, thalamus, amygdala, hippocampus, caudate and insula. Relative to the withdrawal condition, smoking decreased rsFC in the ACC and the medial orbital-frontal cortex.

Indirect evidence that nicotine inhibits activity in the ACC comes from cue studies. The SH model predicts that cue-induced craving and neural activation will be inhibited in the presence of nicotine because nicotine inhibits the craving network. Consistent with the SH model, using functional MRI, Lim *et al*. found neural activation in the ACC when smoking cues were presented during abstinence, but not when cues were presented after smoking [53]. Likewise, Brody *et al*. did not see activation in the ACC with functional MRI when smoking cues were presented soon after subjects had smoked [54]. 

One mechanism by which nicotine might inhibit neural activity is through the activation of nAChRs located on presynaptic GABAergic terminals. Behavioral sensitization to nicotine is associated with increased GABA transmission from the nucleus accumbens to the ventral pallidum [69]. 

Another possible mechanism would be through dopamine. Nicotine increases dopaminergic output from the ventral tegmental area (VTA) [70]. Dopaminergic projections from the VTA to the prefrontal cortex appear to be inhibitory and this inhibition appears to be mediated by D1 receptors [70]. This could explain why smoking has been associated with decreased neural activity (or inhibition of activation) in the frontal cortex in several studies [53,54,66,67,68]. Brody *et al.* demonstrated that changes in the left ventral pallidum and left ventral caudate nucleus/nucleus accumbens dopamine release correlated with craving reduction, and suggested that dopamine release in these areas is associated with decreased craving [71]. Along these lines it is interesting to note that a single dose of haloperidol, a drug that blocks dopamine receptors, has been shown to increase nicotine intake in smokers [72]. This suggests that blocking dopamine receptors disinhibits craving. While much more research is needed, the literature supports the plausibility that nicotine has inhibitory actions.

Brain areas that are activated by nicotine would be among the possible suspects for involvement in the Craving Inhibition System. Notably, the cerebellum showed increased rCBF in response to smoking in two PET studies [66,67]. The cerebellum has reciprocal connections to the ACC and other areas of the frontal and prefrontal cortices [67]. In both PET studies, smoking simultaneously increased activity in the cerebellum while decreasing activity in the ACC. 

To summarize, a number of studies using different paradigms and measures indicate that the ACC and areas of the frontal cortex become activated during craving and exposure to smoking cues. A few studies, using functional MRI, PET and rsFC indicate that nicotine administration inhibits activity in these structures, and this is associated with a reduction in craving. The literature provides initial support for one of the central tenets of the SH model, that nicotine inhibits activity in neural networks that are responsible for generating craving. 

### 3.6. During Abstinence the Withdrawal-Related Adaptations and Tolerance-Related Adaptations Will Activate the Craving Generation System Resulting in Craving (Figure 4, Figure 5, Figure 6)

Consistent with an opponent process model [73,74], the SH model postulates that withdrawal produces a reaction opposite that of nicotine. However, whereas reinforcement models hold that pleasure centers in the brain are activated by stimulant drugs and left hypoactive during withdrawal, the SH model holds that nicotine suppresses the Craving Generation System and withdrawal results in spontaneous activation of the same. We are aware of only two published studies that shed light on this issue. 

Wang *et al*. used arterial spin labeled perfusion MRI to identify neural activity correlated with withdrawal-induced cravings [68]. Fifteen smokers were imaged at rest under conditions of withdrawal and satiety. Consistent with the SH model’s prediction that withdrawal would be associated with increased spontaneous neural activity in structures that are linked to craving, withdrawal was associated with increased rCBF in the ACC, medial orbital-frontal cortex, and left orbital-frontal cortex. The intensity of craving during withdrawal was predicted by rCBF increases in the ACC, right orbital-frontal cortex, right dorsolateral prefrontal cortex, occipital cortex, ventral striatum/nucleus accumbens, thalamus, amygdala, bilateral hippocampus, left caudate and right insula. 

Cole *et al.* used rsFC MRI to examine smokers during satiated and withdrawal conditions. Compared to the satiated state, smokers in withdrawal showed increased rsFC in regions involved in the default mode network [75]. Craving correlated with increased rsFC between the default mode network (which includes the ACC and prefrontal cortex) and the left precuneus (*r* = 0.75), right angular gyrus (*r* = 0.82), and left superior parietal/motor cortex (*r* = 0.83) [75]. 

The authors have initial unpublished data on 8 smokers and 10 nonsmokers from an ongoing study that demonstrates that rsFC in smokers in withdrawal is stronger than that of nonsmokers. Our data also show significant increases in rsFC between the ACC and other structures during withdrawal that correlates with the severity of craving (ACC-superior frontal cortex, *r* = 0.74; ACC-precuneus, *r* = 0.75; ACC-putamen, *r* = 0.77; ACC-inferior parietal cortex, *r* = 0.77). A potential role for an involvement of neuroadaptation in this process is suggested by our data showing changes in structural integrity and structural connectivity in white matter tracts connecting the ACC and superior frontal cortex that also correlate with the intensity of craving.

To summarize, data from these studies suggest that activity in brain circuits involving the ACC increases spontaneously during withdrawal (in the absence of smoking cues) and correlates with the severity of craving. This data is in alignment with the SH model and provides a theory-based explanation for the activation of craving networks in the absence of both the drug and drug cues.

### 3.7. The Tolerance-Related Adaptations Are Irreversible. An Ex-Smoker’s Brain Does Not Return to Its Nicotine-Naïve State (Figure 7)

Investigators tested the prediction that brain changes produced by nicotine are permanent by conducting studies in which rats were sacrificed after different periods of abstinence from nicotine [76,77,78]. The authors concluded that “commensurate with the sensitization-homeostasis model”, adolescent and adult rats show “persistent changes after nicotine exposure” involving serotonergic systems and cell signaling [76,79]. Observing that nicotine self-administration in rats resulted in lowered reward thresholds for at least 36 days after the discontinuation of nicotine, Kenny and Markou postulated that enduring nicotine-induced adaptations may persist in the brains of smokers [80]. Much more research is needed to evaluate the long-term impact of nicotine.

### 3.8. If Tolerance-Related Adaptations Are Irreversible, Successful Smoking Cessation Will Require the Development of Abstinence-Related Adaptations Which Suppress Craving. Continued Abstinence Rests on a Dynamic Equilibrium between Tolerance-Related Adaptations that Stimulate Craving and Opposing Abstinence-Related Adaptations that Suppress Craving (Figure 7)

When ex-smokers lapse they can experience withdrawal-induced craving after the effect of the initial cigarette wears off. This implies that the mechanism that causes withdrawal symptoms remains intact through years of abstinence. If *tolerance-related adaptations* persist for years after smoking cessation, why doesn’t the withdrawal syndrome drag on for decades? According to the SH model, abstinence must trigger the development of *abstinence-related adaptations* that counter the *tolerance-related adaptations*. This is plausible as neural adaptations appear after withdrawal from cocaine [81,82]. 

Brain-derived neurotrophic factor (BDNF) is a protein molecule that has many functions, among which is the stimulation of neuroplasticity [83,84,85]. In humans, two studies have linked specific alleles of the BDNF gene to nicotine dependence [86,87]. A human post-mortem study linked smoking with the expression of BDNF genes in the ventral tegmental area (VTA), concluding that “enduring plasticity within the VTA may be a major molecular mechanism for the maintenance of smoking addiction” [88]. Bhang *et al.* measured plasma BDNF levels at 4 weeks and 12 weeks after smoking cessation in 19 smokers. They reported a significant increase of plasma BDNF at both time points [89]. Kim *et al.* compared plasma BDNF levels in 20 smokers and 20 nonsmokers [90]. BDNF levels were lower among smokers than nonsmokers. BDNF levels were again assessed 2 months after smoking cessation in 12 smokers. At 2 months abstinence, BDNF levels had tripled over baseline values obtained for the same individuals while they were still smoking (1.3 ng/mL *vs.* 0.42 ng/mL). BDNF values in 2-month abstinent smokers were significantly higher than in the nonsmoking controls (1.3 ng/mL *vs.* 0.78 ng/mL), indicating that BDNF levels that were initially low in comparison to those of nonsmokers overshot the levels observed in nonsmokers and had not yet returned to normal after 2 months of abstinence. 

To test the prediction that the cessation of nicotine triggers new changes in the brain, investigators used a rat model of nicotine addiction [76,79,91]. Nicotine was administered to rats for 17 days. Some animals were sacrificed during nicotine administration, some during the acute withdrawal period, and others after different lengths of abstinence. These rats were compared to saline controls. About 3 months after withdrawal from nicotine there were changes in serotonergic systems that were not present during nicotine administration or during early withdrawal. These changes were slow to develop and were evident long after the discontinuation of nicotine [76,91]. While it is not known if these changes are relevant to addiction, the data support the principle that withdrawal from nicotine can prompt the delayed development of persistent changes in the brain. The reinforcement paradigm of addiction does not predict brain remodeling after smoking cessation.

If the *tolerance-related adaptations* persist for many years, the development of neuroadaptations that quiet withdrawal may be critical to cessation. A person’s chances of success with smoking cessation may depend in part on how effectively their brain can produce adaptations that counter the *tolerance-related adaptations*. A physiologic determinant to success at smoking cessation is consistent with recent genetic studies aimed at identifying genes that facilitate the ability to maintain abstinence from smoking [92]. Clusters of gene variants were identified that are present more frequently in successful quitters. The genes identified encode proteins spanning a wide variety of biological functions such as cell adhesion, transcription, receptors and enzymes. These results make sense if neuroplasticity is crucial to cessation. 

No data establish the existence of *abstinence-related adaptations*. However, data indicating that smoking cessation boosts BDNF levels, and animal studies showing changes in brain physiology that appear only after the discontinuation of nicotine, suggest that it is plausible that smoking cessation could trigger neuroplasticity.

### 3.9. When a Long-Abstinent Smoker Slips and Smokes a Cigarette, the Presence of Nicotine Again Disrupts Homeostasis by Suppressing Craving (Figure 9). As the Abstinence-Related Adaptations Also Suppress Craving, Their Presence Augments the Disruption Caused by Nicotine. To Restore Homeostasis, the Brain Dismantles the Abstinence-Related Adaptations (Figure 10). The Lapsed Smoker Will First Experience an Immediate Suppression of Craving Produced by Nicotine, Followed Later by a Resurgence of Craving as the Dismantling of the Abstinence-Related Adaptations Leaves the Withdrawal-Related Adaptations Unopposed, Restoring the Initial State of Addiction. The Intensity of Withdrawal-Induced Craving Triggered by Smoking a Single Cigarette Might Be Nearly as Severe as that Experienced by the Smoker When They Had Quit Smoking. This Causes a Lapse to Turn into a Relapse

The SH model provides an explanation for why a former smoker who lapses by smoking a single cigarette has a 95% probability of relapse [93]. According to the SH model, smoking a cigarette will have an immediate effect of reducing craving, with a delayed effect of a resurgence of craving and great difficulty in sustaining abstinence. An experiment by Shadel *et al.* directly tested these predictions [94]. Smokers were asked to remain abstinent indefinitely. After 2 days of abstinence, half of the subjects were instructed to smoke 2 cigarettes while the other half were not allowed to smoke. The 2 groups were compared in relation to craving and their ability to maintain abstinence. The group that had smoked 2 cigarettes reported an immediate reduction in craving. However, the following day, craving in that group rebounded, surging above that experienced by the controls. Smoking the 2 cigarettes hastened relapse. While these data support the theory, this is the only study to date that has addressed these particular predictions of the SH model. Future studies will help to clarify these predictions.

## 4. Updates to the SH model

Recent advances in our understanding of the development of nicotine addiction allow us to update the SH model.

### 4.1. The Latency to Withdrawal

The central role in addiction of the latency to withdrawal (LTW) was not understood when the SH model was developed. The LTW is the length of time the smoker can go without nicotine before withdrawal symptoms emerge. Ten years ago it was assumed that withdrawal was triggered by nicotine levels falling below a certain threshold [95], and that the LTW was therefore determined by the serum half-life of nicotine. Recent evidence indicates that this cannot be true. If the LTW was tied to the half-life of nicotine, all smokers would have a similar LTW, allowing for individual differences in rates of nicotine metabolism. The reported range for the LTW extends from a few minutes to greater than 4 weeks (4 orders of magnitude) [15,96,97], while nicotine metabolism varies only 2–4 fold [98,99]. So the range in the LTW cannot be explained on the basis of nicotine metabolism.

Early in the development of addiction, smokers report that smoking a single cigarette keeps withdrawal symptoms at bay for weeks [15,96,97]. *Based on these reports we extend the SH model by stipulating that nicotine must affect brain function for at least weeks after it has been removed from the brain*. Nicotine has effects on dopamine, glutamate, GABA, serotonin, endorphins and norepinephrine [100]. Based on evidence that nicotine alters long-term potentiation and gene expression, it might be expected that its actions would endure after the nicotine is gone. This appears to be the case. Rats treated with nicotine injections for 7 days showed increased basal overflow of dopamine in the nucleus accumbens more than 48 h later [101]. The first dose of nicotine in naïve rats lowers the response threshold in neurons for 28 days, and stimulates increased release of norepinephrine in the hippocampus that starts 2 weeks after the exposure and continues out to a month after exposure [27,102]. Behavioral sensitization to nicotine persists for at least a month after the last dose [103]. Prenatal exposure to nicotine resulted in elevated baseline levels of the early protein c-fos in the infralimbic cortex in the offspring during adolescence [104]. One dose of nicotine given to adolescent rats produces behavioral changes that are present a month or more later [33,34]. While it is not known what action of nicotine might be responsible for the fact that a single cigarette can keep withdrawal symptoms at bay for weeks, evidence is emerging that the impact of nicotine on the brain persists long after it has been cleared. 

It has been established through case histories that the LTW places an outside limit on how far apart smokers can comfortably space their cigarettes [46,97]. Each cigarette resets the timer on the LTW, analogous to hitting the snooze button on an alarm clock. Empirical data indicate that at the onset of addiction the LTW may exceed one week, but over time the duration of relief from withdrawal that is afforded by smoking a cigarette shortens [46,97]. The LTW can shorten to as little as a few minutes, prompting individuals to smoke seemingly without interruption. About half of adolescent smokers are aware that their LTW has been shrinking [97]. 

The shortening of the LTW represents a form of tolerance because subsequent doses of nicotine have less impact on forestalling withdrawal. The shortening of the LTW is the only form of tolerance to nicotine that has been demonstrated to correlate with addiction severity [97,105]. The original SH model postulated that tolerance involved neuroadaptations that opposed the inhibitory affect of nicotine on the craving generation system. *We update the SH model by proposing that tolerance-related adaptations work, not by decreasing the magnitude of nicotine’s inhibitory effect on the craving generation system, but rather by decreasing the duration of its effect, and this manifests clinically in the shortening of the LTW*.

According to case histories, as the LTW shortens, smokers feel compelled to smoke at more frequent intervals. The progressively shortening LTW appears to explain the smooth trajectory in escalating smoking frequency observed in longitudinal studies [106,107], first in terms of the number of smoking days per month until smoking occurs on a daily basis, and then in terms of the number of cigarettes smoked per day [12]. The early onset of addiction combined with the shortening of the LTW may offer an explanation for why smoking 2 cigarettes per week at age 12 increases the chances of progressing to heavy smoking as an adult with an odds ratio of 174 [49]. The progressive shortening of the LTW would make the escalation of smoking inevitable unless cessation was achieved. 

To our knowledge, no studies demonstrate that smokers adjust their smoking frequency to maintain a level of nicotine in their blood. When the LTW has shrunk to a few hours or less, smokers will maintain blood levels of nicotine because their short LTW requires them to smoke at frequent intervals, but that is not why they are smoking frequently. A logical interpretation of the literature might suggest that smokers require a “hit” of nicotine at intervals determined by their LTW, and that the blood level of nicotine at the time is irrelevant. This would imply that in animal models of nicotine addiction it would be preferable to employ intermittent dosing rather than continuous exposure from implantable osmotic pumps.

### 4.2. The Levels of Physical Dependence

While the SH model acknowledges a role for psychological dependence and cue-reactivity [108], it is primarily a physical dependence model as it holds that the primary factor directing the smoker’s actions is the need to suppress a withdrawal-induced desire to smoke whenever the effect of nicotine wears off. In the original SH model, this desire to smoke is simply termed “craving”, but our recent studies reveal that smokers actually experience 3 quantitatively and qualitatively distinct forms of a withdrawal-induced desire to smoke that we have given operational definitions as “wanting”, “craving” and “needing” [46,109]. 

“Wanting” as a withdrawal symptom is described as a mild desire to smoke that is short-lived and easily ignored. It comes on after a characteristic interval of abstinence in the absence of any smoking cues and does not intrude upon the smoker’s thoughts. “Craving” is a stronger urge to smoke that is more persistent and difficult to ignore. By definition, craving intrudes upon smokers’ thoughts, interrupting their concentration. It feels like their brain is telling them that it is time to smoke [46]. “Needing” is an intense and urgent desire to smoke that is unpleasant and unremitting. Smokers cannot concentrate on anything other than the urgency of needing to smoke, and understand that they will not feel and function normally until they obtain relief by smoking. Each of these symptoms appears after a characteristic interval of abstinence and in the absence of smoking cues. 

Multiple lines of clinical evidence indicate that wanting, craving and needing as withdrawal symptoms appear in the same developmental sequence in all smokers [46,110,111]. In other words, early in the process of addiction, smokers experience only wanting when they are in withdrawal, later they will experience wanting followed by craving when they are in withdrawal, and finally they will experience wanting, followed by craving, followed by needing when they are in withdrawal. We developed and validated a measure of the level of physical dependence based upon the smoker’s position along this sequence [46,109,110,111,112,113]. We call this a measure of physical dependence because it is based entirely on an assessment of the smoker’s subjective withdrawal symptoms. 

The fact that physical dependence appears to develop in a set sequence in all smokers implies that withdrawal-related adaptations must also develop in a set sequence in all smokers. If the SH model is correct in attributing the withdrawal-induced desire to smoke to withdrawal-related adaptations, and if physical dependence develops in the same sequence in all smokers, it might be possible to correlate the development of physical dependence to changing neural features in the brain. To test this theory, we correlated smokers’ level of physical dependence to a measure of the physical structure of the brain. Before describing the results of that study it would be helpful to review prior studies of brain structure in smokers. 

Using MRI many investigators have found structural differences between the brains of smokers and nonsmokers [114,115,116,117,118]. Fractional anisotropy (FA) is an MRI measure of the complexity of white matter structure. Hudkins *et al*. reported that FA in the ACC white matter bundle correlated inversely with scores on the Fagerström Test for Nicotine Dependence (FTND), a measure of the severity of physical dependence (*r* = −0.64) [28]. Zhang *et al*. reported an inverse correlation (*r* = −0.52) between FA and FTND score in prefrontal cortex white matter in highly dependent smokers only [115]. In a small study, Paul *et al*. reported a non-significant inverse correlation (*r* = −0.58) between FA and FTND in the corpus callosum [29]. In these cross-sectional studies, the authors speculated that FA findings might reflect a pre-existing “heritable difference between smokers and nonsmokers” [115], nonspecific “toxicity” [29], or the disruption of the “trophic effects of acetylcholine” on neurodevelopment [30]. None of the authors raised the possibility that structural differences might represent neuroadaptations related to addiction. 

Returning now to our unpublished pilot study, we found that FA in the dorsal ACC bundle correlated with our measure of the progression of physical dependence at *r* = −0.85 (*p* < 0.05). Together, these four studies suggest that in adult smokers, a decline in FA in white matter structures tracks the progression of physical dependence, and this suggests a possible role for neuroadaptation. The picture is complicated by the observation that smoking appears to increase FA in adolescents, while in adults, FA decreases with increasing pack-years of smoking [28,30]. Hudkins *et al.* have suggested that smoking increases FA in adolescent smokers and then decreases FA in adult smokers [28]. This would help to explain a paradox: FA decreases with advancing pack years of smoking in adults and yet smokers have higher FA than nonsmokers [28,29,30]. An increase in FA triggered by smoking during adolescence would boost FA above the level of nonsmokers, but FA would then decline during adulthood. This explanation raises the question as to why smoking would appear to have opposite effects on FA in adolescents and adults if it is a pre-existing condition or results from toxicity. 

We suggest here that the relevant consideration is not the age of the subjects in these studies but where they sit in relation to the onset of addiction. Adolescent smokers are earlier in the progression of dependence than the adult smokers in these studies. These studies lend to speculation that as addiction initially develops there is a neuroplastic process that increases FA, and in established addiction, there is a different neuroplastic process that decreases FA. The SH model predicted that the onset of dependence involves neuroadaptations that increase stimulatory inputs to the Craving Generation System (*withdrawal-related adaptations*). This prediction was based on animal studies showing that nicotine administration resulted in increased dendritic length and spine density in the nucleus accumbens and cingulate cortex [119]. These effects might be expected to increase FA by increasing structural complexity. The increase in FA at the onset of smoking in adolescents might possibly reflect the development of *withdrawal-related adaptations* that initiate addiction. 

What addiction-related neuroplastic process might explain the subsequent decline in FA in established smokers that correlates so strongly with the advancement of physical dependence? According to the updated SH model, *tolerance-related adaptations* are responsible for the shortening of the LTW. As tolerance develops, the LTW shortens and the number of cigarettes smoked per day increases. The original SH model postulated that *tolerance-related adaptations* might involve the pruning of inhibitory inputs to the Craving Generation System. Pruning might be expected to decrease tissue complexity with a resulting decline in FA. The decline in FA in established smokers might therefore reflect the development of *tolerance-related adaptations*. The idea that FA in the ACC and the LTW are both related to *tolerance-related adaptations* is supported by the observation that both the FA in the ACC (*r* = −0.58) and the LTW (−0.53) are inversely correlated with the number of cigarettes smoked per day [28,97]. If FA in the ACC is not tied to the LTW, how else can its correlation with the number of cigarettes smoked per day be explained?

### 4.3. What Happens after Cessation?

The original SH model indicated that *tolerance-related adaptations* persist after smoking cessation. Our updated model implicates the *tolerance-related adaptations* for the shortening of the LTW. Therefore, if tolerance-related adaptations persist, so too must the shortening of the LTW. The impact of long-term abstinence on the LTW can be evaluated indirectly by determining the frequency at which smokers feel compelled to smoke immediately after a relapse. 

To study the impact that a prolonged period of abstinence has on the number of cigarettes smoked per day upon the resumption of smoking, 2 surveys of adult smokers were conducted [120]. In the first survey, subjects who had been abstinent for 6 months reported resuming smoking at a mean of 34% of their baseline lifetime peak consumption, while those in a replication study resumed smoking at 48% of baseline peak rates. These data suggest that periods of abstinence up to 6 months result in a lengthening of the LTW as smokers were not required to smoke as frequently as they had prior to quitting smoking. However, continued abstinence beyond 6 months had a negligible additional effect as smokers who had been abstinent from 2 to 29 years resumed smoking at a mean of 39% of their pre-quit peak lifetime cigarette consumption. Had their brains returned to their original state, these smokers would not feel compelled to smoke at 39% of their prior levels but would be content smoking a cigarette every few weeks as they had when they first started smoking. These data suggest that there are two processes that contribute to determining the LTW. One is reversible and the other is not.

EEG tests also suggest that the brains of smokers do not revert to their original state after smoking cessation. Neuhaus *et al.* demonstrated dysfunctional frontal lobe activation on EEG in both smokers and ex-smokers as compared to nonsmokers [121]. Differences between ex-smokers and nonsmokers might indicate that nicotine produces persistent changes in the brain, or they might reflect pre-existing conditions that predisposed the ex-smokers to take up smoking. 

## 5. Errors in the Original SH Model

One stipulation of the SH model that has not withstood the test of time is the idea that smokers eventually reach a point where they strive to maintain a steady state of nicotine in their systems as postulated by Jarvik [122]. Based on nicotine’s half-life, it is estimated that a person would have to smoke at least 5 cigarettes per day to maintain a minimum threshold of nicotine in the blood throughout the day [95]. More than a dozen studies have reported withdrawal symptoms in individuals who smoke fewer than 5 cigarettes per day [4,5,6,7,8,11,13,16,123,124,125,126,127,128,129,130]. As nondaily smokers commonly experience nicotine withdrawal symptoms, but do not maintain a minimum threshold of nicotine in the blood, it is evident that the presence of withdrawal symptoms does not require smokers to achieve or maintain a minimum threshold of nicotine in the blood. To the contrary, all available evidence indicates that the prime determinant of smoking frequency is not the half-life of nicotine, but rather the latency to withdrawal.

In attempting to explain why nondaily smokers could go as long as they could between cigarettes, the original SH model suggested that acetylcholine might act on up-regulated and sensitized receptors to provide brain stimulation similar to that obtained by nicotine. Given recent data on how quickly receptor upregulation can resolve [19] and how long addicted smokers can go between cigarettes, this mechanism now seems less probable than one based on downstream effects of nicotine on other neurotransmitters and gene expression. 

## 6. Conclusions and Future Directions

We hope that this updated model of SH will continue to provide a framework for addressing the potential role of neuroadaptation in nicotine addiction through the lens of a clinically-grounded model that provides a theoretical basis for withdrawal-related, tolerance-related and abstinence-related neural adaptations. Some of the important tenets of the SH model are now well supported by the literature. The idea that nondaily smokers have symptoms of addiction, once thought impossible, appears to be generally accepted. While more research would be welcome, published studies are very consistent in their documentation of the rapid onset of addiction symptoms in nondaily smokers, and the experience of nicotine withdrawal in nondaily and light daily smokers. These findings should be extended to other drugs of abuse to determine how quickly addiction develops in relation to other drugs.

There is now evidence that physical dependence to nicotine develops in a set sequence of symptoms in all smokers. A validated measure of the level of physical dependence is now available for use in imaging studies [109,110]. The ability to quantify progression through the levels of physical dependence provides neuroscientists with an unprecedented opportunity to correlate the clinical progression of dependence with neuroadaptive changes in brain structure or function. The levels of dependence have a distinct advantage over traditional dependence measures for imaging studies because the levels of dependence are known to occur in a set sequence while this is not true of items on the FTND. This sequential perspective can facilitate the interpretation of findings.

The SH theory places much more emphasis than any other theory of addiction on the desire to smoke that is generated by withdrawal. Indeed, “craving” as a withdrawal symptom was eliminated in DSM-IV [131]. The idea that craving involves neural networks is not unique to the SH model and now appears to be generally accepted. As we have reviewed, there is now emerging evidence that nicotine inhibits activity in craving circuits while smoking cues have the opposite effect. 

The SH model emphasizes the inhibitory properties of nicotine. Based on the reinforcement paradigm, many studies have focused on areas that are stimulated by addictive drugs. Much more research is needed on areas that are inhibited by nicotine. Timing may be critical in this line of research. If nicotine is withheld too long in an addicted subject, the effects observed may represent relief from withdrawal rather than a primary action of nicotine. There is little consistency in the observed effects of an acute dose of nicotine in neuroimaging studies [132,133], and this might reflect the lack of control for withdrawal states on the one hand, and recent exposure to nicotine on the other. Similarly, the SH model indicates that it is important to control for recent nicotine intake in cue studies as smoking prior to imaging or cue exposure could inhibit cue reactivity within the craving generation system. On the other hand, if a subject has been abstinent too long, the craving generation system might be activated spontaneously by withdrawal and the presentation of cues might have little additional impact. 

Researchers should remain cognizant of the LTW and accommodate it in their study designs and selection of subjects. When designing MRI studies, researchers must remember that smokers differ up to 10,000 fold in their LTW. Because subjects differ in their LTW, the time since the last cigarette does not provide a good measure of their state of withdrawal. After the same interval of abstinence, smokers with different LTWs will be in different stages of withdrawal. By setting high minimum daily cigarette consumption requirements for participation in their studies prior investigators have unwittingly restricted their subject pool to smokers with very short LTWs. Uniformity in the LTW among subjects may limit the power to detect important correlations. For similar reasons it would be important for investigators to include subjects from all levels of physical dependence. The LTW and the levels of physical dependence are important correlates to measure and manipulate in imaging studies. 

When the SH model was proposed, the prevailing paradigm held that addictive drugs of all types stimulate brain reward centers while withdrawal leaves these areas hyporesponsive [74]. The SH model posited that nicotine would have the opposite effect, in that withdrawal from nicotine would produce spontaneous activation of craving circuits. Limited experimental data now show that nicotine withdrawal is accompanied by increased neural activity, or coordination of activity, that correlates with craving. Although we do not know how to tie it in with the sensitization-homeostasis model, it is curious to note that nicotine also appears to be unique among addictive drugs in that withdrawal is associated with a persistent lowering of reward thresholds, while drugs like cocaine and heroin have the opposite effect [80]. Much more research is needed to explore how nicotine withdrawal affects brain activity. 

The observation that the LTW can change over time by a factor of 10,000 suggests an important addiction-related neuroadaptation. If not for this process, addicted smokers might be satisfied with smoking 2 cigarettes per week over a lifetime of addiction. We speculate that identifying the tolerance process that is responsible for shrinking the LTW will present a future challenge for our field. 

Given that physically dependent smokers can go weeks between cigarettes [46,97], and that the nAChR blocker mecamylamine does not trigger nicotine withdrawal in humans [134], it appears unlikely that levels of nAChR occupancy are important to withdrawal. We postulate that it must be some downstream effect of nicotine that is responsible for keeping withdrawal at bay. We would welcome an animal model of physical dependence that develops through small intermittent exposures [135], as it does in humans and in which mecamylamine challenge does not precipitate withdrawal.

The Sensitization-Homeostasis model describes three distinct forms of neuroadaptations with specific functions and properties. *Withdrawal-related adaptations* are responsible for the rapid development of addiction and withdrawal-induced craving. They develop quickly and resolve quickly with smoking cessation. *Tolerance-related adaptations* typically develop over many years and are responsible for the shortening of the latency to the onset of withdrawal craving. They do not resolve after smoking cessation and are responsible for relapse. *Abstinence-related adaptations* develop after smoking cessation and quiet withdrawal-related craving. They appear critical to a smoker’s ability to remain abstinent. They are dismantled quickly when an abstinent smoker lapses by smoking a cigarette.

The SH model is a simple physiologic model that explains a wide range of phenomena without reference to reward or reinforcement. Nicotine addiction differs in important ways from other forms of drug addiction. We should entertain the prospect that reward may be why people use nicotine, but it may not be the mechanism by which nicotine causes addiction. 
